# Systemic versus local responses in melanoma patients treated with talimogene laherparepvec from a multi-institutional phase II study

**DOI:** 10.1186/s40425-016-0116-2

**Published:** 2016-03-15

**Authors:** Howard L. Kaufman, Thomas Amatruda, Tony Reid, Rene Gonzalez, John Glaspy, Eric Whitman, Kevin Harrington, John Nemunaitis, Andrew Zloza, Michael Wolf, Neil N. Senzer

**Affiliations:** Rutgers Cancer Institute of New Jersey, 195 Little Albany Street, Room 2004, New Brunswick, NJ 08901 USA; Minnesota Oncology, Fridley, MN USA; University of California San Diego Medical Center, La Jolla, CA USA; University of Colorado Cancer Center, Aurora, CO USA; UCLA Jonsson Comprehesive Cancer Center, Los Angeles, CA USA; Carol G. Simon Cancer Center, Morristown, NJ USA; The Institute of Cancer Research/Royal Marsden NIHR Biomedical Research Centre, London, UK; Mary Crowley Cancer Research Centers, Dallas, TX USA; Amgen Inc., Thousand Oaks, CA USA

**Keywords:** Herpes virus, Immunotherapy, Melanoma, Oncolytic virus, Talimogene laherparepvec, T-VEC

## Abstract

**Background:**

We previously reported that talimogene laherparepvec, an oncolytic herpes virus encoding granulocyte-macrophage colony-stimulating factor (GM-CSF), resulted in an objective response rate of 26 % in patients with advanced melanoma in a phase II clinical trial. The response of individual lesions, however, was not reported. Since talimogene laherparepvec is thought to mediate anti-tumor activity through both direct tumor cytolysis and induction of systemic tumor-specific immunity, we sought to determine the independent response rate in virus-injected and non-injected lesions.

**Methods:**

Fifty patients with stage IIIC or IV melanoma were treated with talimogene laherparepvec in a multi-institutional single-arm open-label phase II clinical trial. In this study patients were treated until a complete response was achieved, all accessible tumors disappeared, clinically significant disease progression, or unacceptable toxicity. This report is a post hoc analysis of the systemic effects of talimogene laherparepvec in injected lesions and two types of uninjected lesions—non-visceral lesions and visceral lesions.

**Results:**

Eleven of 23 patients (47.8 %) had a ≥ 30 % reduction in the total burden of uninjected non-visceral lesions, and 2 of 12 patients (16.7 %) had a ≥ 30 % reduction in the total burden of visceral lesions. Among 128 evaluable lesions directly injected with talimogene laherparepvec, 86 (67.2 %) decreased in size by ≥ 30 % and 59 (46.1 %) completely resolved. Of 146 uninjected non-visceral lesions, 60 (41.1 %) decreased in size by ≥ 30 %, the majority of which (44 [30.1 %]) completely resolved. Of 32 visceral lesions, 4 (12.5 %) decreased in size by ≥ 30 %, and 3 (9.4 %) completely resolved. The median time to lesion response was shortest for lesions that were directly injected (18.4 weeks), followed by uninjected non-visceral lesions (23.1 weeks) and visceral lesions (51.3 weeks), consistent with initiation of a delayed regional and systemic anti-tumor immune response to talimogene laherparepvec.

**Conclusions:**

These results support a regional and systemic effect of talimogene laherparepvec immunotherapy in patients with advanced melanoma.

## Background

The therapeutic landscape for the treatment of metastatic melanoma has been changing dramatically over the last few years driven by progress in the clinical application of targeted therapy and tumor immunotherapy [1]. Immunotherapy has gained considerable attention, in part because of high response rates with some monotherapy and combination therapy regimens (such as immune checkpoint inhibitors), evidence of overall survival benefit in randomized clinical trials, and the durability often obtained with immunotherapy agents [2–4]. There is now emerging evidence that lymphocyte-predominant tumors, characterized with high numbers of effector T cells (and a weighted effector T cell/regulatory T cell ratio), may be more susceptible to immunotherapy and strategies to increase the lymphocytic infiltration to tumors are a high priority for improving clinical responses to immunotherapy.

Talimogene laherparepvec, an oncolytic herpes simplex virus type 1 (HSV-1) [5], was gene modified to elicit increased selectivity and rescue replication in tumors as well as improved tumor antigen presentation. It contains deletions of the neurovirulence factors ICP34.5 and a factor that blocks peptide loading onto the major histocompatibility complex (MHC) called ICP47. These changes reduce the pathogenicity of the virus. Talimogene laherparepvec also contains an insertion of the human granulocyte-macrophage colony-stimulating factor (GM-CSF) gene sequence at the deleted ICP34.5 coding sequence sites to enhance systemic immune response [6]. We previously reported that in a multi-institutional, single-arm, open-label, phase II clinical trial, intralesional injection of talimogene laherparepvec resulted in an ORR of 26 % in patients with stage IIIC and stage IV melanoma. The majority of responding patients had a durable remission in both injected and uninjected non-visceral lesions (with MART-specific CD8+ T cells observed in both subsets) [7] including visceral sites for 7–31 months, indicating a possible systemic effect [8]. Recently, a randomized phase III clinical trial confirmed this objective response rate and demonstrated an especially high durable response rate for patients with stage IIIB/C and IV M1a disease [9]. This trial led to the U.S. Food and Drug Administration (FDA) approval of talimogene laherparepvec for local treatment of unresectable cutaneous, subcutaneous, and nodal lesions in patients with melanoma recurrent after initial surgery. Talimogene laherparepvec has not been shown to improve overall survival in patients with visceral metastases [10]. Talimogene laherparepvec has been associated with a tolerable safety profile and provides a new strategy for directly killing tumor cells, promoting local lymphocyte infiltration, and use in combination regimens to improve clinical responses to tumor immunotherapy. The ability of talimogene laherparepvec to mediate systemic clinical anti-tumor activity compared to exerting a more local effect on lymphocyte responses has been previously described [8] but not fully quantified.

This report is a post hoc analysis from a phase II study in melanoma of potential systemic effects of talimogene laherparepvec based on an analysis of individual virus-injected lesions and two types of uninjected lesions—non-visceral lesions and visceral lesions. We sought to determine the independent response rate as well as median time to lesion response in these different types of lesions. The results provide insight into the level of systemic activity possible with talimogene laherparepvec in patients with melanoma and provide support for combination studies in which talimogene laherparepvec-induced anti-tumor immunity can be enhanced with other agents known to expand or inhibit suppression of antigen-specific T cells.

## Methods

### Study design

The full details of the overall design and methods for the phase II clinical trial have been previously reported [8]. Briefly, patients received up to 8 doses of talimogene laherparepvec over a 15-week period. Talimogene laherparepvec was administered at an initial dose of 10^6^ plaque-forming units (PFU)/mL and injected into 1 or more skin or subcutaneous lesions (up to 4 mL total). Subsequent doses began 3 weeks after the first dose and consisted of talimogene laherparepvec at 10^8^ PFU/mL (up to 4 mL total) every 2 weeks. The volume of talimogene laherparepvec delivered to each lesion depended on the size of the lesion measured on the day of virus administration. In this study, at least 1 lesion was to be left uninjected to assess systemic response. Uninjected lesions were to be at least 5 cm from the nearest injected lesion. Tumor responses were derived based on modified Response Evaluation Criteria in Solid Tumors (RECIST) version 1.0.

If indications of biological activity were observed after the initial 8 doses (stable disease or better, inflammatory response in an uninjected non-visceral lesion, and/or injection site reaction) and patients did not have evidence of clinically symptomatic disease progression or unacceptable toxicity, treatment could be continued for an additional 16 doses unless the investigator determined that another therapy was appropriate.

The protocol was approved by the site investigational review boards (see Acknowledgements) and by the U.S. FDA under an investigational new drug application. All patients provided written informed consent.

### Response analysis in injected and uninjected lesions

To evaluate the systemic effect of talimogene laherparepvec (ie, beyond local effects in injected lesions), the following endpoints were evaluated by lesion type: patient incidence of overall lesion-type response, incidence of lesion response, maximum decrease in tumor burden (individual lesion and overall by lesion type) and the time to individual lesion response. Analyses of systemic effects were based on baseline or new measurable lesions from patients in a pre-planned safety analysis population. Evaluable lesions were defined as those with measurements recorded at ≥ 2 visits (with a measurable lesion size noted in the earliest assessment). All analyses were based on investigator assessment of lesion measurements and lesion injection status.

### Statistical analysis

For this study descriptive statistics were used. Measurable lesion characteristics were displayed by lesion type (injected, uninjected non-visceral, and visceral), and the patient incidences of measurable lesion locations were tabulated by lesion type. Overall lesion-type burden was calculated as the sum of the longest diameters of all lesions of the same type at a study visit. The patient incidence of overall lesion response was reported as the proportion of patients with a ≥ 30 % decrease in overall lesion burden by lesion type. Graphical presentations of the maximum decrease in overall lesion-type burden were produced using waterfall plots. The maximum decrease in the size of individual lesions (based on the longest diameter) was categorized (>0 %, ≥ 30 %, 100 %) and presented by lesion type. The incidence of lesion response was reported as the proportion of lesions with a ≥ 30 % decrease in size. Graphical presentations of the maximum decrease in baseline or new measurable lesions were produced using waterfall plots.

The time to lesion response by lesion type was analyzed using the Kaplan-Meier method for all evaluable lesions of the same lesion type. Kaplan-Meier estimates of event quartiles and the corresponding 95 % confidence intervals, when estimable, were based on a sign test [11]. Time to lesion response was evaluated from the date of the baseline measurement to the date of first response (for new lesions, the first appearance of the lesion was considered the baseline measurement).

## Results

### Measurable lesion characteristics

Evaluable patients for the lesion-level analyses (patients with at least 1 lesion with measurements at ≥ 2 visits) and overall lesion-type burden analyses (patients with ≥ 2 visits with measurement of all lesions of the same type) are summarized in Table 1. Of the 50 patients who received talimogene laherparepvec injected into a lesion in the phase II clinical trial, 48 patients (96.0 %) also had at least 1 uninjected lesion (Table 1). This included 26 patients (52.0 %) with only uninjected non-visceral lesions at baseline and 22 patients (44.0 %) with at least 1 visceral lesion at baseline. Of 400 baseline or new lesions, 306 were evaluable (i.e., had measurements at ≥ 2 visits), including 146 (47.7 %) uninjected non-visceral lesions and 32 (10.5 %) visceral lesions). The most common anatomic locations of uninjected, non-visceral lesions were lymph nodes, cutaneous metastases, or subcutaneous nodules. Visceral lesions were most commonly located in the lungs and liver (Table 2).

**Table 1:** Patient characteristics of measurable lesion type in talimogene laherparepvec study

	Any Measurable Lesion	Evaluable^a^ Lesion	Evaluable for overall lesion- type burden^b^
	n (%)	n (%)	n (%)
Any	50 (100)	47 (100)	37 (100)
At least 1 baseline uninjected lesion	48 (96.0)	41 (87.2)	35 (94.6)
Baseline uninjected, non-visceral only	26 (52.0)	23 (48.9)	23 (62.2)
At least 1 visceral lesion at baseline	22 (44.0)	15 (31.9)	12 (32.4)

Denominators are the numbers of patients

^a^Evaluable indicates at least 2 assessments with valid measurements per investigator

^b^Evaluable indicates at least 2 visits with non-missing overall lesion-type burden for each respective patient subgroup, or overall tumor burden for “Any”

All patients received at least one dose of talimogene laherparepvec

**Table 2:** Anatomic location of measurable uninjected lesions in talimogene laherparepvec study

	Any	Evaluable^a^
	n (%)	n (%)
Any uninjected non-visceral, number of patients (%)	49 (100)	42 (100)
Any non-visceral, number of patients (%)	48 (98.0)	39 (92.9)
Head/Neck, Front, number of lesions (%)	2 (4.1)	1 (2.4)
Head/Neck, Back	1 (2.0)	1 (2.4)
Head/Neck, Right	4 (8.2)	4 (9.5)
Head/Neck, Left	4 (8.2)	4 (9.5)
Trunk, Front	13 (26.5)	10 (23.8)
Trunk, Back	6 (12.2)	5 (11.9)
Lower Limb, Right	5 (10.2)	5 (11.9)
Lower Limb, Left	8 (16.3)	8 (19.0)
Upper Limb, Right	4 (8.2)	3 (7.1)
Upper Limb, Left	2 (4.1)	1 (2.4)
Right Hand, Palm	1 (2.0)	1 (2.4)
Right Hand, Back	1 (2.0)	1 (2.4)
Groin	2 (4.1)	2 (4.8)
Lymph node, specify	21 (42.9)	16 (38.1)
Other	16 (32.7)	8 (19.0)
Any visceral, number of patients (%)	23 (46.9)	15 (35.7)
Eye, number of lesions (%)	1 (2.0)	0 (0.0)
Brain	3 (6.1)	0 (0.0)
Lung	17 (34.7)	12 (28.6)
Gastrointestinal Tract	3 (6.1)	1 (2.4)
Kidney	3 (6.1)	0 (0.0)
Adrenal	4 (8.2)	1 (2.4)
Liver	8 (16.3)	4 (9.5)
Pancreas	3 (6.1)	2 (4.8)
Spleen	4 (8.2)	0 (0.0)

Denominator is the total number of patients. Patients may have multiple lesions

^a^Evaluable indicates at least 2 assessments with valid measurements

All patients received at least one dose of talimogene laherparepvec

### Patient incidence of overall lesion-type response

Of the 23 evaluable patients with baseline uninjected non-visceral disease, 11 patients (47.8 %) had a ≥ 30 % reduction in the total burden of uninjected non-visceral lesions (Table 3). Of the 12 evaluable patients with visceral disease, 2 patients (16.7 %) had a ≥ 30 % reduction in the total burden of visceral lesions. In general, the observed response rate per investigator was consistent with the rate of overall lesion-type response (derived from the tumor burden change within a lesion subset; see Table 3). The overall tumor response rate for patients with only uninjected non-visceral lesions was 43.5 % per investigator and 47.8 % based on the change in the total patient burden of uninjected non-visceral lesions. The overall tumor response rate for patients with visceral-only lesions was 16.7 %, both per investigator and based on the change in the total patient burden of visceral lesions.

**Table 3:** Summary of talimogene laherparepvec responses by lesion-type

		Overall tumor response		Overall lesion-type response
	Any	OR	CR	Yes
	n (%)	n (%)	n (%)	n (%)
Any	37 (100)	14 (37.8)	8 (21.6)	15 (40.5)
At least 1 baseline uninjected lesion	35 (100)	12 (34.3)	6 (17.1)	13 (37.1)
Baseline uninjected non-visceral only	23 (100)	10 (43.5)	5 (21.7)	11 (47.8)
At least 1 visceral lesion at baseline	12 (100)	2 (16.7)	1 (8.3)	2 (16.7)

Denominator is the total number of patients in corresponding patient lesion-type subgroup

Objective response (*OR*), and complete response (*CR*) as per investigator-reported responses. Overall lesion response was reported as the proportion of patients with a ≥ 30 % decrease in overall lesion burden. OR was evaluated by modified RECIST.

Overall lesion type response: max decrease ≥ 30 % in overall lesion-type burden from baseline in patient lesion-type subgroup and in total tumor burden for “Any”

All patients received at least one dose of talimogene laherparepvec

### Incidence of lesion response

**Fig. 1:**
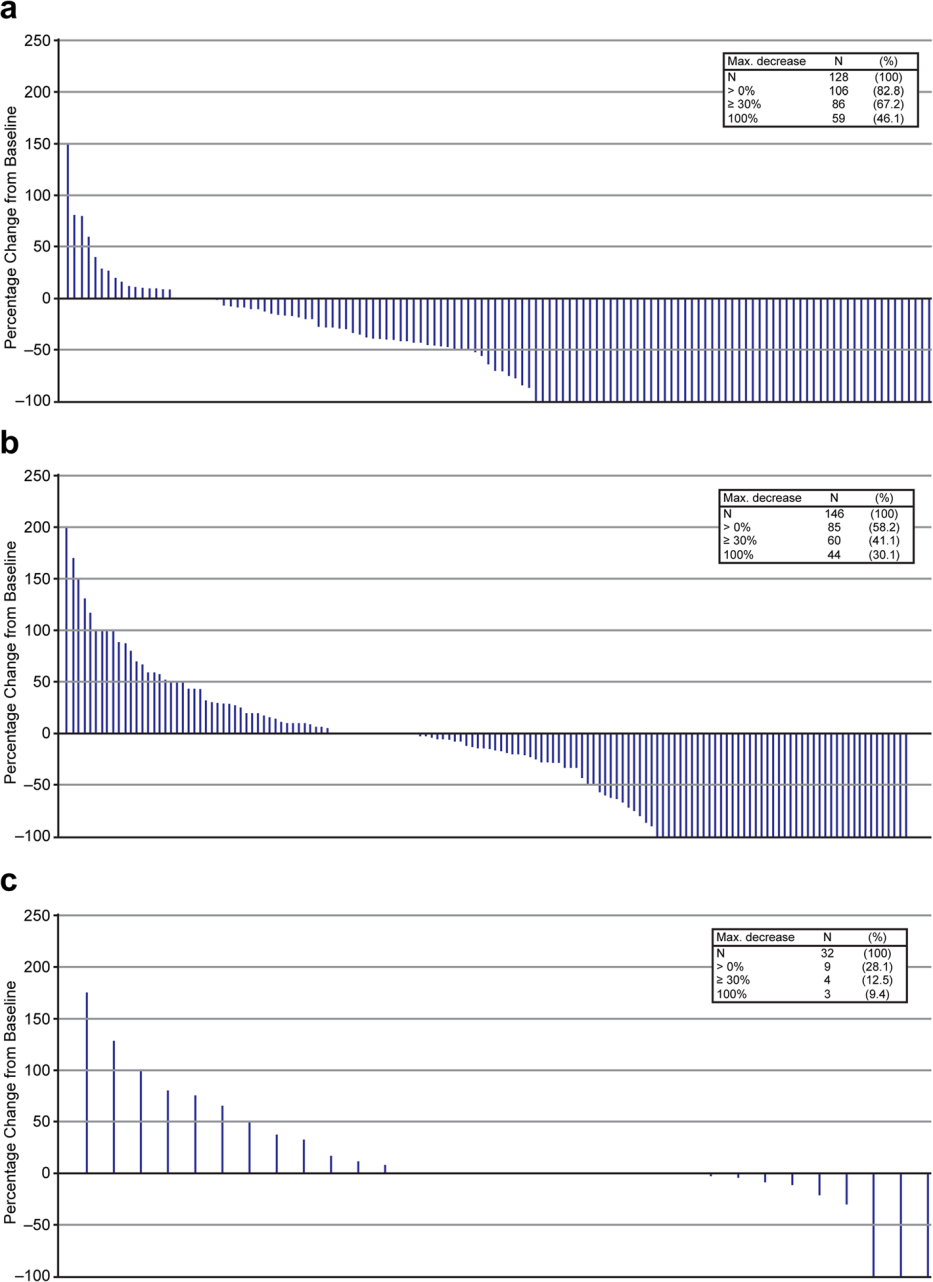
Maximum percent decrease in evaluable lesions: (**a**) Injected lesions, (**b**) Uninjected non-visceral lesions, and (**c**) Visceral lesions. Lesion measurements per investigator. Evaluable indicates at least 2 assessments with valid measurements. Uninjected lesion indicates baseline or new lesions never known to be injected. Safety analysis set consisted of the patients who received at least one dose of study therapy

The waterfall plot of the maximum decrease of individual lesions is shown in Fig. 1a. Among 128 evaluable lesions directly injected with talimogene laherparepvec, 86 (67.2 %) decreased in size by ≥ 30 %, and 59 (46.1 %) completely resolved (Fig. 1a). Responses were also observed in baseline and new uninjected lesions, including both non-visceral and visceral lesions as shown in the waterfall plot for baseline and new measurable lesions that appeared during the course of study treatment (Fig. 1b). Of 146 uninjected non-visceral lesions, 60 (41.1 %) decreased in size by ≥ 30 %, the majority of which (44 [30.1 %]) completely resolved. The 44 uninjected non-visceral, non-visceral baseline or new lesions that completely resolved were in 6 of 24 patients with a baseline or new uninjected non-visceral lesion that had a CR. Of 32 visceral lesions, 4 (12.5 %) decreased in size by ≥ 30 %, the majority of which (3 [9.4 %]) completely resolved (Fig. 1c). The 3 baseline or new visceral lesions that completely resolved were in 1 of 12 patients with baseline or new visceral lesions that had a CR.

### Time to lesion response

**Fig. 2:**
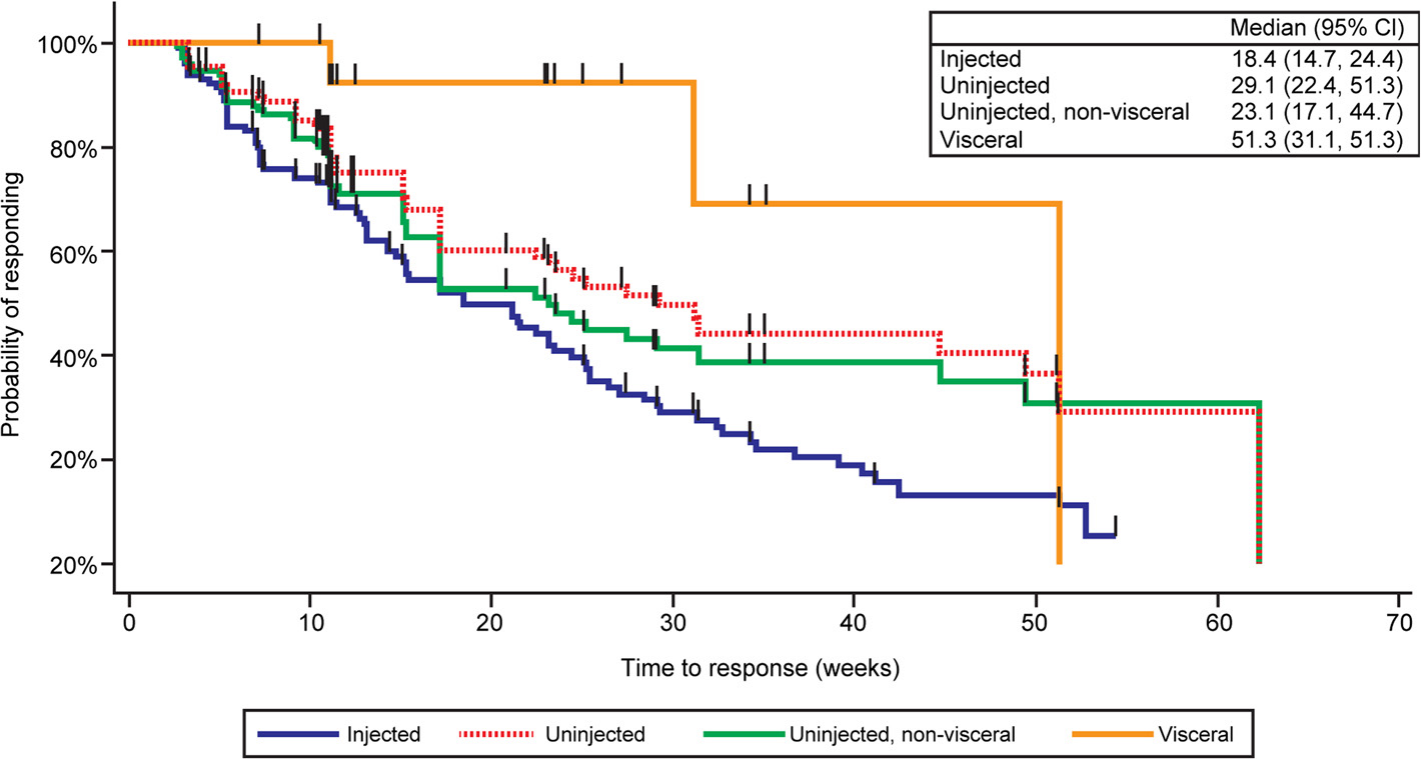
Lesion level time to response (Kaplan-Meier survival curves) for talimogene laherparepvec for injected, all uninjected, uninjected non-visceral, or visceral lesions in the phase II clinical trial of talimogene laherparepvec in patients with stage IIIC or IV melanoma. Safety analysis set consisted of patients who received at least one dose of study therapy

The median time to lesion response (baseline or new lesions) is shown in Fig. 2 and was shortest for lesions that were directly injected (18.4 weeks) compared to all uninjected non-visceral lesions (29.1 weeks). On further analysis of uninjected lesions, uninjected non-visceral lesions responded at a median of 23.1 weeks compared to visceral lesions which responded at a mean of 51.3 weeks. These results are consistent with initiation of a delayed regional and systemic anti-tumor immune response to talimogene laherparepvec.

## Discussion

Talimogene laherparepvec is an oncolytic herpes virus encoding GM-CSF and was designed to exert anti-tumor effects through both a direct oncolytic effect in injected lesions and through induction of systemic anti-tumor immunity. In a multi-institutional phase II clinical trial, an objective response rate of 26 % was reported and evidence of systemic anti-tumor immunity was also seen with MART-specific CD8+ T cells observed in both injected and uninjected lesions of responding patients [8]. In this post hoc analysis, we confirmed the total tumor burden reduction in both types of uninjected lesions—uninjected non-visceral and visceral lesions—as evidence of both regional and systemic immune response elicited by talimogene laherparepvec. However, the regional response rate was higher than systemic responses when analyzing both total tumor burden and individual tumor lesions. While this could relate to a slowly expanding T cell response primed close to the site of virus injection, it may also suggest that direct injection and oncolysis are important for maximizing therapeutic response of individual tumor lesions. Interestingly, the majority of lesions that had  ≥ 30 % reduction in size went on to complete resolution, and this was seen across all types of lesions—injected, uninjected non-visceral, and visceral lesions. This supports the possibility that talimogene laherparepvec can successfully induce an effective systemic anti-tumor response with only a local, intratumoral, injection and immunotherapy mechanism as an important component of the mechanism of action for talimogene laherparepvec.

Numerous attempts to incite or modulate systemic immune responses via intralesional immunotherapy have been reported over the last 50 years. One of the earliest studies dates back to the 1970s when Dr. Donald Morton at the John Wayne Cancer Institute reported the effect of intralesional Bacillus Calmette-Guerin (BCG) in a cohort of patients with unresectable melanoma [12], although a subsequent randomized phase III trial failed to prove the systemic efficacy of this approach in 2004 [13]. Nevertheless, a number of phase I/II studies with intralesional cytokines (eg, GM-CSF, IL-2, interferons), viruses (eg, HSV-1, Coxsackievirus, Poxviruses), and plasmids (eg, Allovectin) have been conducted but their use has remained sporadic, and they have not progressed to become established treatments [14–17]. Talimogene laherparepvec was designed to increase both oncolytic effects through modulation of the HSV-1 vector for tumor-specific HSV-1 and to generate enhanced systemic response through deletion of the viral ICP47 gene and expression of GM-CSF [5]. The herpes ICP47 gene product blocks antigen presentation and normally prevents immune recognition of viral antigens. When this gene product is deleted, it is hypothesized that both viral-specific and pre-formed tumor-specific antigens are more likely to be presented by major histocompatibility complexes (MHC) and thus, generate T cell responses. GM-CSF expression within the local tumor microenvironment following injection of talimogene laherparepvec serves as a patient-specific, *in situ*, method of maturing dendritic cells and, hence priming local cytotoxic T cell responses. These results are consistent with other GM-CSF expressive immunotherapies [18–21] supporting systemic clinical effect. Further studies need to be done to better define whether viral- or tumor antigen-induced T cell repertoires are responsible for tumor rejection with talimogene laherparepvec and other oncolytic viruses.

In this study, we found a higher response rate of talimogene laherparepvec in the control of uninjected non-visceral lesions compared to the control of visceral lesions. This could relate to local spread of virus, which could mediate oncolytic effects on uninjected lesions, or it could be due to intrinsic differences in the therapeutic effectiveness of talimogene laherparepvec in various sites. Effector T cells are likely primed close to the injected tumors, likely in regional draining lymph nodes, and must be released into the systemic circulation, where they must survive and traffic to other sites of tumor growth. Expression of certain chemokine receptors and adhesion molecules on T cells are essential in this homing process. T cells primed in one location generally result in homing to a similar tissue compartment [22]. For example, native herpes simplex virus infection preferentially primes lymphocytes that express CCR4 and CCR10, chemokine receptors that promote trafficking to the skin [23]. In contrast, gut-tropic T cells express a high level of the chemokine receptor CCR9 and migrate preferentially to the lamina propria of the small intestine [24]. Dendritic cells (DCs) play an instructive role in efficient T cell priming and influence their function in a tissue-specific fashion by imparting variable chemokine receptor expression following priming [25]. DCs are also responsible for the imprinting of tissue-specific homing potential [26, 27]. Thus, DCs from the injected lesion may specifically prime effector T cells targeting tumors in similar locations, which could explain why uninjected non-visceral lesions were more susceptible to rejection after talimogene laherparepvec was injected into other skin-associated tumors. Further, the role of viral infection, local inflammation, and delivery of GM-CSF on the formation of resident T cells responding to tumor antigens is not fully understood. Resident memory CD8+ T cells (T_RM_) primed in the skin afford global protection within the skin, but do not circulate to other sites [28–30]. Their generation in this context, along with decreased induction of tissue-circulating effector memory T cells (T_EM_) or secondary lymphoid tissue-circulating central memory T cells (T_CM_), could serve as an explanation for the increased resolution of uninjected non-visceral lesions compared to visceral lesions.

Another possible explanation for the better response of uninjected non-visceral lesions may be that these lesions are simply closer to TVEC-injected lesions compared to visceral lesions and it may be easier and faster for activated T cells to migrate to these lesions due to the shorter distance. An alternative explanation involving distance from the injection lesion may be that uninjected non-visceral lesions are likewise infected during infection of nearby injected lesions. However, animal studies in which Newcastle Disease Virus was injected into one lesion failed to detect virus in contralateral lesions within the same tissue [31]. Further investigation is needed to better understand how tumors are rejected with talimogene laherparepvec treatment and the nature and activity of tumor-reactive T cells need to be more fully defined. Future clinical studies will examine the impact of direct injection of talimogene laherparepvec into visceral tumors and this may provide an opportunity to better understand if local injection can overcome the more limited visceral lesion response observed with injection of accessible skin and soft tissue lesions, which has characterized most oncolytic virus clinical trials to date. Another strategy in clinical development is to combine oncolytic viruses with other T cell promoting immunotherapy agents, most notable T cell and other immune checkpoint inhibitors. These agents may enhance the activation status of talimogene laherparepvec-primed T cell responses and would be expected to increase the therapeutic activity at both a local and systemic level.

Overall, we observed that in 12 out of 23 patients with evaluable visceral lesions, only 2 out of 12 patients had evaluable visceral lesion responses and only 4 out of 32 total evaluable visceral lesions had an objective response. In the pivotal phase III clinical trial, a higher response rate was observed in patients with unresectable stage IIIB/C and IV M1a disease, suggesting this population may be better suited for treatment with talimogene laherparepvec. A major limitation of our study was the relatively small sample size, which may have obscured the significance of the systemic response elicited by talimogene laherparepvec. An evaluation of lesion responses in patients treated on the much larger phase III clinical trial may help resolve this issue [9]. Other limitations of this study include the need to rely on investigator assessment, different methods of lesion measurement (e.g. clinical calipers for skin and soft tissue lesions vs. CT-guided imaging for visceral lesions), lack of immune response data and time bias since different lesion types were measured at different time points and immunotherapy may result in a delayed kinetics of therapeutic response. Nonetheless, these data provide some insight into the mechanism of talimogene laherparepvec activity and help identify appropriate patients for treatment with talimogene laherparepvec.

## Conclusions

In conclusion, the ability of talimogene laherparepvec to initiate regional and systemic anti-tumor activity is supported by responses in both uninjected non-visceral and some visceral melanoma lesions. The time to lesion response was shortest for lesions that were directly injected, followed by uninjected non-visceral lesions and visceral lesions, consistent with initiation of a delayed systemic anti-tumor immune response following talimogene laherparepvec treatment. Given the recent FDA approval of talimogene laherparepvec for the treatment of melanoma, further studies are needed to better understand how talimogene laherparepvec mediates anti-tumor activity. New studies are planned to evaluate therapeutic responses through direct injection of visceral tumors and combinations with other T cell enhancing immunotherapy agents. These studies should provide new insight into the optimal patient selection for talimogene laherparepvec treatment and will likely guide further improvements in the therapeutic effectiveness of oncolytic viruses as a new class of drugs for the treatment of cancer.

## Abbreviations

BCG: Bacillus Calmette-Guerin
CR: Complete response
DCs: Dendritic cells
FDA: Food and Drug Administration
GM-CSF: Granulocyte-macrophage colony-stimulating factor
HSV-1: Herpes simplex virus type 1
IL-2: Interleukin-2
MHC: Major histocompatibility complex
ORR: Objective response rate
PFU: Plaque-forming unit
RECIST: Response Evaluation Criteria in Solid Tumors
T_RM_: Resident memory CD8+ T cells
T_EM_: Effector memory T cells
T_CM_: Central memory T cells
